# Distribution and biogeography of *Sanguina* snow algae: Fine‐scale sequence analyses reveal previously unknown population structure

**DOI:** 10.1002/ece3.6772

**Published:** 2020-09-14

**Authors:** Shawn P. Brown, Avery E. Tucker

**Affiliations:** ^1^ Department of Biological Sciences University of Memphis Memphis TN USA; ^2^ Center of Biodiversity Research University of Memphis Memphis TN USA

**Keywords:** biogeography, ITS2, minimum entropy decomposition, populations, *Sanguina*, snow algae

## Abstract

It has been previously suggested that snow algal species within the genus *Sanguina* (*S. nivaloides* and *S. aurantia*) show no population structure despite being found globally (*S. nivaloides*) or throughout the Northern Hemisphere (*S. aurantia*). However, systematic biogeographic research into global distributions is lacking due to few genetic and no genomic resources for these snow algae. Here, using all publicly available and previously unpublished *Sanguina* sequences of the Internal Transcribed Spacer 2 region, we investigated whether this purported lack of population structure within *Sanguina* species is supported by additional evidence. Using a minimum entropy decomposition (MED) approach to examine fine‐scale genetic population structure, we find that these snow algae populations are largely distinct regionally and have some interesting biogeographic structuring. This is in opposition to the currently accepted idea that *Sanguina* species lack any observable population structure across their vast ranges and highlights the utility of fine‐scale (sub‐OTU) analytical tools to delineate geographic and genetic population structure. This work extends the known range of *S. aurantia* and emphasizes the need for development of genetic and genomic tools for additional studies on snow algae biogeography.

## Graphical abstract

It has been previously suggested that snow algae within the genus *Sanguina* are globally cosmopolitan both in terms of occurrence and population structure. Using all publicly available and previously unpublished *Sanguina* sequences of the Internal Transcribed Spacer regions, we investigate whether this purported global population ubiquity of *Sanguina* is supported by evidence. Here, in direct opposition to the currently accepted cosmopolitan nature of these algae populations, we demonstrate global population structure and highlight the utility of fine‐scale (sub‐OTU) analytical tools to delineate geographic and genetic population structure.

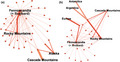

## 1 INTRODUCTION

Snow algae are a diverse group of organisms that have adapted to live within and on snow ecosystems. Most snow algal taxa belong to the Chlamydomonadales (Chlorophyta) but they can also be found within the Euglenophyta, Cryptophyta, and Chrysophyta among others, albeit with scant representation (Hoham & Remias, 2020). Snow phycological systems have historically been underexplored, with relatively few active researchers (Hoham, 1980; Hoham & Duval, 2001; Kol, 1968; Weiss, 1983), but recently there has been a renaissance of sorts where global investigations into these algae and associated communities have grown extensively (Anesio, Lutz, Chrismas, & Benning, 2017; Brown & Jumpponen, 2019; Brown, Olson, & Jumpponen, 2015; Brown, Ungerer, & Jumpponen, 2016; Davey et al., 2019; Hamilton & Havig, 2017, 2020; Krug, Erlacher, Markut, Berg, & Cernava, 2020; Lutz et al., 2016; Lutz, McCutcheon, McQuaid, & Benning, 2018; Lutz, Prochazkova, Benning, Nedbalova, & Remias, 2019; Müller, Bleiss, Martin, Rogaschewski, & Fuhr, 1998; Procházková, Leya, Křížková, & Nedbalová, 2019; Procházková, Remias, Řezanka, & Nedbalová, 2018; Remias, Procházková, Holzinger, & Nedbalová, 2018; Remias, Procházková, Nedbalová, Andersen, & Valentin, 2020; Segawa et al., 2018; Takeuchi, 2013; Yoshimura, Kohshima, & Ohtani, 1997). While snow algae are diverse, perhaps the most well known are algae that form red blooms in late season open field snows caused by the recently established genus *Sanguina* (Procházková et al., 2019) [previously assigned to *Chlamydomonas* cf. *nivalis*] which currently comprises two delineated species, *S. nivaloides* and *S. aurantia*.

We know relatively little about these *Sanguina* spp. due to our current inability to culture *Sanguina,* which would facilitate controlled experimentation and genomic characterization, but they are presumed to consist of green haploid vegetative cells. These cells take advantage of a dynamic layer of water that has a fluctuating solid and liquid phase where peak levels of liquid water occur during the summer/spring. During that time, abiotic and biotic deposits are more readily solubilized and made accessible to the vegetative cells (Jones, 1999). These flagellated haploid cells are thought to reproduce asexually, and when nutrients, primarily nitrogen and phosphorous, become limited, haploid gametes fuse and produce a diploid hypnozygote with thick cell walls that produce vast quantities of the secondary carotenoid astaxanthin and its fatty ester derivatives, leading to red snow coloration (Gorton, Williams, & Vogelmann, 2001; Müller et al., 1998). These hypnozygotes are resting cysts that allow overwintering, and upon snow ablation, meiosis occurs. As snows continue to melt, vegetative cells will also encyst to protect the organisms and facilitate oversummering. *S. nivaloides* is globally distributed and has been found on every continent (Brown & Jumpponen, 2019; Hoham & Remias, 2020; Novis, 2002; Procházková et al., 2019; Segawa et al., 2018) but much about the basic biology, metabolism, and reproduction strategies of this genus remains unresolved. Further, despite numerous morphological examinations (Kol, 1968; Leya, 2013; Procházková et al., 2019; Remias, 2012; Remias, Lütz‐Meindl, & Lütz, 2005; Weiss, 1983) and molecular characterizations (Brown & Jumpponen, 2019; Brown et al., 2016; Krug et al., 2020; Lutz et al., 2016; Procházková et al., 2019; Segawa et al., 2018), we know very little about global dispersal capabilities of these algae, which is likely to be an important factor in structuring landscape population assemblies.

Given that *Sanguina nivaloides* is cosmopolitan and has been found across the globe where perennial snows are present, and *S. aurantia* has an apparent circumpolar and alpine distribution across the Northern Hemisphere (Procházková et al., 2019), it is surprising that no population structure, even across intercontinental distances, has been seen to occur (Brown & Jumpponen, 2019; Procházková et al., 2019). Recent work has suggested very little sequence variation within targeted loci (Brown & Jumpponen, 2019; Brown et al., 2016; Procházková et al., 2019; Segawa et al., 2018), and no observed local isolation by distance can be found (Brown et al., 2016). Further, Procházková et al. (2019) conducted the most detailed to date investigations into population and genetic structure using a haplotype approach for the ITS2 region and failed to find distinct population structure across the ranges of these *Sanguina* species, although they did identify serval *S. nivaloides* haplotypes suggesting some genetic differences. Two recent publications expand on the apparent lack of population structure of *Sanguina nivaloides*. Procházková et al. (2019) wrote the following:

Our data showed a cosmopolitan distribution of *S. nivaloides* in alpine and polar snowfields in both hemispheres, which supports the theory of a trans‐equatorial dispersal of microbes (Hodač et al., 2016). No population structure was detected when analysing the ITS2 rDNA data, as there was no phylogeographic signal. Metagenomic analyses have shown red pigmented snow algae to be cosmopolitans based on the analysis of partial sequences of the 18S rRNA gene (Lutz et al., 2016) as well as of the ITS2 rDNA (Segawa et al., 2018).

Further, Brown and Jumpponen (2019) similarly came to this following conclusion:

These core algae … highlight two main points of discussion: (1) common snow algae are extremely conserved globally with nearly identical ITS2 sequences found across vast distances and across years, and (2) we know very little about the global genetic diversity or dispersal patterns of these snow algae.

Taken together, this suggests that genetic variation within populations may be indistinguishable globally and even may be near‐identical and all accessions in the global sequence repositories seemingly support this assertion (Brown & Jumpponen, 2019; Brown et al., 2016; Lutz et al., 2018). This brings up important but currently unanswered questions: how are these algae dispersed and how do they colonize snows? It seems unlikely that global populations of *Sanguina* have active gene flow over vast distances or trans‐equatorial dispersal capabilities given that long‐distance aerial transport is presumed unlikely outside extreme weather events (Brown & Jumpponen, 2019). This lack of population structure globally may be an artifact of sequence representation as most investigations into *Sanguina* molecular ecology target the 18S ribosomal RNA gene (SSU) or the Internal Transcribed Spacer region 2 (ITS2) of the rRNA gene operon. The 18S is generally highly conserved, which may preclude fine‐scale demarcation of algal taxa (Lutz et al., 2018) but ITS regions are hypervariable and can be readily used to demarcate algal species (An, Friedl, & Hegewald, 1999; Brown et al., 2016). It is surprising, given the hypervariable nature of ITS regions, that we would observe near‐identical sequences globally, but similar observations from the fungal literature suggest that on rare occasions, some species have extreme conservation in ITS sequences across vast geographic separations (Hughes, Morris, & Segovia, 2015; Hughes, Tulloss, & Petersen, 2018). This potential hypersimilarity of rRNA regions may explain these observed patterns in *Sanguina,* but we do not have enough non‐rRNA sequence data as of yet to determine whether this is an aberration or if these species are in fact globally hypersimilar. Here, we harvest all publicly available ITS2 sequences for both *S. nivaloides* and *S. aurantia* to investigate global population structures using a minimum entropy decomposition approach to examine if *Sanguina* spp. do in fact have homogeneous population structure across their ranges.

## 2 MATERIALS AND METHODS

To investigate global population structure of *Sanguina*, we use a minimum entropy decomposition (MED; Eren et al., 2015) framework to create sensitive unsupervised sequence partitions (MED nodes) based on local base pair entropy. MED iteratively partitions gene marker data into homogeneous nodes (MED nodes) based on only information‐rich nucleotide base pairs, thereby omitting stochastic variation from the obtained nodes. This has been demonstrated to provide sensitive, but informative, separation of closely related sequences and strains. To do so, we analyzed all available and verified ITS2 sequences at the time of analysis from *Sanguina* species from GenBank, SRA, and supplemental information from associated publications. We chose to analyze the ITS2 region as opposed to 18S or other gene targets because ITS2 has the most available data, and ITS regions have great potential for species‐level population analysis for algae (An et al., 1999). We gathered the following Sanger sequences: 56 sequences from Segawa et al. (2018) collected from Alaska (USA), Svalbard (Norway), and Antarctica; 48 sequences from Procházková et al. (2019) from Austria, Italy, Slovakia, Switzerland, Norway, Colorado (USA), Argentina, and Antarctica; 29 sequences (Brown, unpublished using the primers ITS1‐ITS4) from Lyman Basin, Washington (USA; 48°10′21″N, 120°53′50″W, 1,880 m asl) and Niwot Ridge, Colorado (USA; 40°02′56″N, 105°34′51″W, 3,514 m asl). Further, we gathered locus‐targeted Illumina MiSeq sequence data: 1,600 sequences (Brown et al., 2016) from Washington (USA) and Colorado (USA); 44,666 sequences (Brown & Jumpponen, 2019) from Finland, Sweden, Norway, and Colorado (USA); and 59,130 sequences (Tucker & Brown, unpublished*;* using fITS7‐ITS4 primers) from Lyman Basin, Washington (USA; 48°10′27″N, 120°53′26″W; 1818 m asl), Mt. Democrat, Colorado (USA; 39°20′38″N, 106°07′45″W, 3,950 m asl) and Medicine Bow Peak, Wyoming (USA, 41°20′45″N, 106°019′50″W; 3,549 m asl). In all, we gathered 105,529 ITS2 sequences. All sequences used were to the best of our knowledge from snows, generally perennial snowfields. To confirm that these sequences were from *Sanguina* snow algae, we extracted the ITS2 region (remove flanking 5.8S and LSU regions) from all sequences using the program ITSx (Bengtsson‐Palme et al., 2013), and MAFFT aligned (Katoh & Standley, 2013) them to create a multiple sequence alignment (MSA). To initially confirm *Sanguina* origin of sequences, all sequences were initially clustered into OTUs using VSEARCH at 3% dissimilarity (Rognes, Flouri, Nichols, Quince, & Mahé, 2016) and representative sequences for these OTUs (see Appendix A1) were queried against GenBank (BLASTn nr/nt) and type sequences for both *Sanguina* species to confirm *Sanguina* identities. This resulted in two retained OTUs —the dominant OTU1 (best match to *Sanguina aurantia*, 96.63% match to accession MK728633.1—38,012 total sequences; 95.65% match to *S. aurantia* type specimen MK728634.1) and OTU2 (best match to *Sanguina nivaloides,* 99.59% match to accession GU117577.1—22,065 total sequences, 99.01% match to *S. nivaloides* type specimen MK728599.1), and remaining sequences were determined to not belong to *Sanguina* and were discarded. Discarded sequences were mainly assigned to the Trebouxiophyceae, other non‐*Sanguina* Chlorophyceae or were poorly matched to any reference taxa. It may be that a few errant sequences not belonging to either target *Sanguina* species may have been included as part of the OTU clustering, but we have no evidence that casts doubt on the veracity of these sequences. These retained OTUs will hereafter be referred to as *S. aurantia* or *S. nivaloides*. All associated retained sequences were collected (Table 1; Appendix S1) and coded by location for *Sanguina* species‐specific MED analyses (*S. nivaloides* and *S. aurantia* were analyzed separately). Some locations were binned to increase sequence representation or based on geographic proximity; Colorado and Wyoming sequences were combined as Rocky Mountains, Finland, Sweden, Norway (including Svalbard) were combined as Fennoscandia (+ Svalbard), and all European samples apart from the Nordic countries were binned as Europe.

**Table 1:** Locations and number of verified *Sanguina* species sequences used for this study

Species	Location	Number of sequences
*S. aurantia*	Fennoscandia (+ Svalbard)	32,094
*S. aurantia*	Rocky Mountains (USA)	424
*S. aurantia*	Cascade Mountains (USA)	5,490
*S. aurantia*	Alaska (USA)	4
*S. nivaloides*	Fennoscandia (+ Svalbard)	5,474
*S. nivaloides*	Europe	21
*S. nivaloides*	Cascade Mountains (USA)	917
*S. nivaloides*	Rocky Mountains (USA)	15,650
*S. nivaloides*	Argentina	1
*S. nivaloides*	Antarctica	2

All aligned sequences for *S. aurantia* and *S. nivaloides* separately underwent minimum entropy decomposition (Eren et al., 2015) to demarcate ecologically relevant operational units (MED nodes) for each species. This yielded 36 MED nodes for *S. aurantia* and 25 MED nodes for *S. nivaloides* (Table S1. Appendix A1). MED node distribution networks were visualized using the program Gephi (v.0.9.2; Bastian, Heymann, & Jacomy, 2009), and cluster analysis (as implemented in the program MED) along with associated visualizations was conducted using Canberra distances on MED Node × Location matrices for *S. nivaloides* and *S. aurantia* separately. Canberra distance (Lance & Williams, 1967) maximizes the effect of differences between samples with many low or zero values which some of our locations have (Table 1) and was calculated using the program MED following Equation 1.

(1) $$D_{\mathrm{Canberra}}=\frac{1}{S_{T}}\sum_{i=1}^{S_{T}}\frac{\left|S_{\mathrm{Ai}}‐S_{\mathrm{Bi}}\right|}{S_{\mathrm{Ai}}+S_{\mathrm{Bi}}}$$

where *S_Ai_* is the abundance of the *i*th MED node in sample A, *S_Bi_* is the abundance of the ith MED node in sample B, and *S_T_* is the total number of MED nodes in samples A and B. Further, Node x Sample matrices were used to test if distributions of MED nodes differed between locations. If these *Sanguina* snow algae possess no or minimal population structure as suggested in the literature, then the composition of MED node distributions should be similar across all locations; *H_0_* = equal proportions of each decomposed MED Node independent of sampling locations. To test distributional differences of node composition, we used *K*‐sample Anderson‐Darling tests (Scholz & Stephens, 1987) using the package *k‐Samples* in R (Scholz & Zhu, 2019). *K*‐sample Anderson‐Darling tests examine if samples are from a common unspecified distribution function, we conducted these tests to determine if locations differ in MED node compositions, and where significantly different, post hoc tests were conducted to examine which samples differ. We used the asymptotic *P‐*value approximation method, and individual tests were corrected for multiple comparisons using a Šidák correction method (α for *S. aurantia* = 0.0085 and for *Sanguina nivaloides* = 0.0034).

## 3 RESULTS

Our collected sequences (Table 1) indicate that *S. aurantia* is only found in the Northern Hemisphere with circumpolar and temperate alpine distributions, which is in line with published reports (Procházková et al., 2019). *Sanguina nivaloides* appears to have a global and bipolar distribution (Procházková et al., 2019; Segawa et al., 2018). However, our current understanding of these species’ ranges is limited due to the paucity of sampling from polar and alpine regions across the globe. These distributions are likely to be expanded when more locations are sampled. Here, we see that the known range of *S. aurantia* (know previously only from Svalbard and Colorado; Procházková et al., 2019) is expanded to include the Cascade Mountains and Alaska. Additional sampling efforts would refine our current rudimentary understanding of these species ranges.

**Figure 1:**
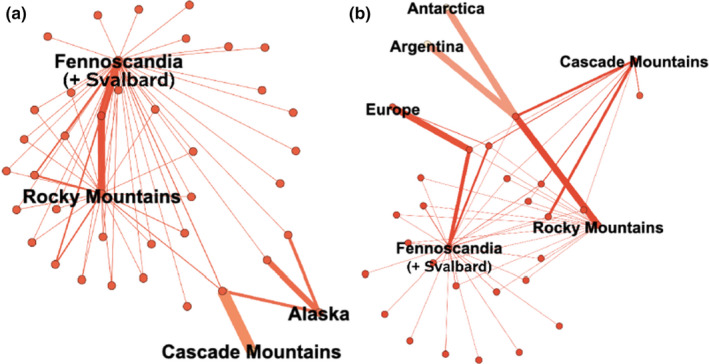
Network visualization of MED nodes for (a) *Sanguina aurantia* and (b) *Sanguina nivaloides* depicting connectivity between sampling locations. Thickness of connecting line is indicative of number of shared MED nodes. Overall, we see high MED node connectivity suggesting similar, but distinct sequences for these snow algae globally. Networks visualized using the program Gephi

**Figure 2:**
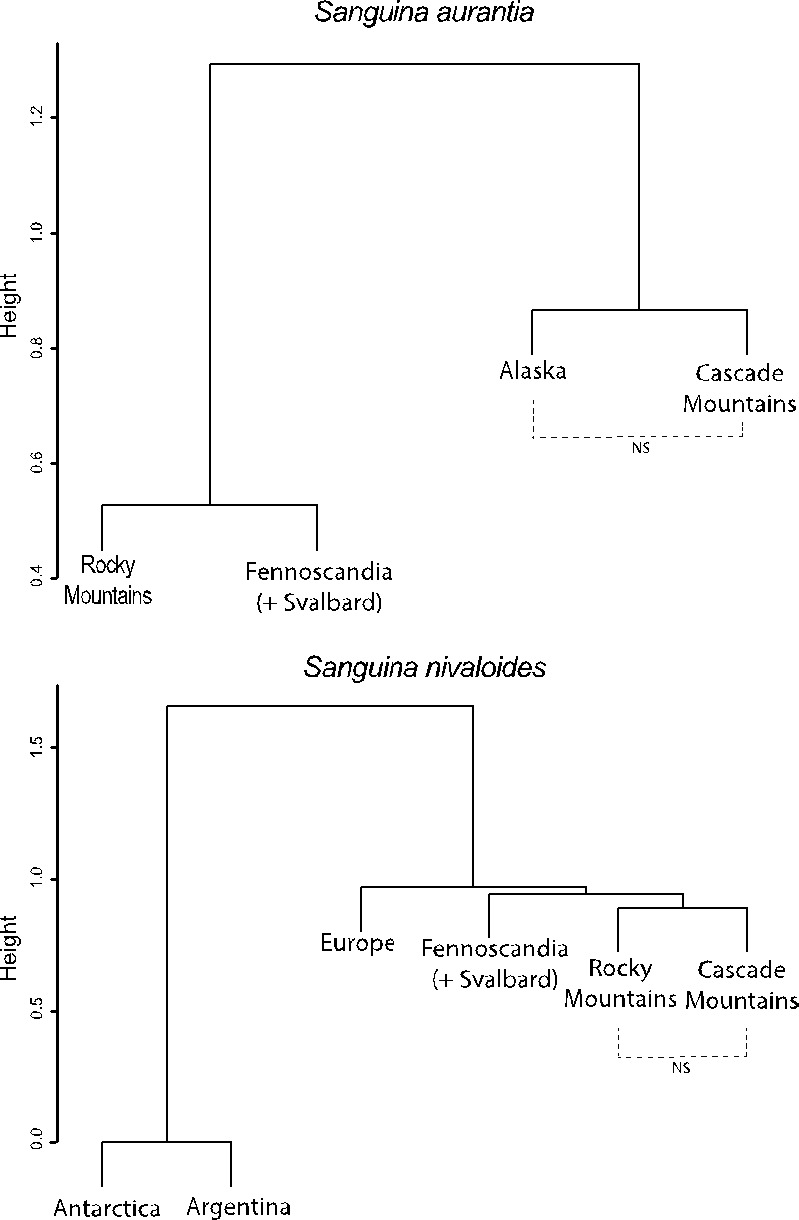
Results of clustering analysis using Canberra distances for *Sanguina aurantia* (top) and *S. nivaloides* (bottom) depicting regional similarity of snow algae populations. Where population structure is indistinguishable, and of sufficient sample size (Table 2) it is indicated with dashed connecting lines and denoted with “NS.”

Overall, there is strong node connectivity within *Sanguina aurantia* and *Sanguina nivaloides* (Figure 1) among locations, indicative of numerous shared MED nodes (closely related individuals) between locations. Even though there were many closely related sequences, we see strong and significant differences in node distributions. Both *S. aurantia* and *S. nivaloides* were seen to have different node composition globally (Figure 2, Table 2; T.AD is the standardized test statistic, T.AD = 61.78, *p* = 5.62 × 10^−40^ and T.AD = 37.44, *p* = 4.15 × 10^−28^ respectively). Post hoc comparisons suggest that there are differences between populations across locations (Table 2) with *S. aurantia* exhibiting different MED node communities for all location comparisons except the Cascade Mountains and Alaska in the United States (T.AD = 0.019, *p* = .349). This indicates that for *S. aurantia*, populations across the Northern Hemisphere are generally distinct with the exception of the Western United States (and potentially Canada, though no Canadian data are available). For *S. nivaloides*, we observe (Table 2) that Antarctic and Argentinean populations are indistinguishable (T.AD = −1.363, *p* = 1.0) as are Antarctica and Europe (T.AD = −0.852, *p* = .895), Argentina and Europe (T.AD = −0.852, *p* = .895), and the Cascade Mountains and the Rocky Mountains (T.AD = 1.572, *p* = .072). Our cluster analysis (Figure 2) suggests that there may be hemispheric segregation, where Antarctic and Argentinian sample populations of *S. nivaloides* are shown to be more similar than to Northern Hemisphere populations. However, Antarctic and Argentinian samples consisted of very few sequences (Table 1), so associated inferences about these distributions should be taken with reasonable skepticism, but given the geographic proximity of the Antarctic and Argentinian samples, we think this is likely a true pattern but more data are needed to confirm.

**Table 2:** Results of k‐Sample‐based Anderson‐Darling (AD) tests of *Sanguina* species MED node distributions with post hoc comparisons between groups

Test	AD test statistic	*p*‐Value
*Sanguina aurantia* – Šidák corrected α = 0.0085		
***All Locations***	***61.78***	***5.62*** × ***10^−^^40^***
***Alaska* versus *Rocky Mountains***	***21.69***	***7.67*** × ***10^−^^10^***
***Alaska* versus *Fennoscandia (+Svalbard)***	***57.94***	***5.33*** × ***10^–^^25^***
Alaska versus Cascade Mountains	0.01898	0.3494
***Rocky Mountains* versus *Fennoscandia (+Svalbard)***	***32.43***	***2.47*** × ***10^−^^14^***
***Rocky Mountains* versus *Cascade Mountains***	***27.14***	***4.03*** × ***10^−^^12^***
***Fennoscandia (+Svalbard)* versus *Cascade Maintains***	***58.97***	***1.97*** × ***10^−^^25^***
*Sanguina nivaloides* – Šidák corrected α = 0.0034		
***All Locations***	***37.44***	***4.16*** × ***10^−^^28^***
Antarctica versus Argentina	−1.363	1
Antarctica versus Europe	−0.8528	0.8952
***Antarctica* versus *Rocky Mountains***	***20.17***	***3.31*** × ***10^−09^*** ^a^
***Antarctica* versus *Fennoscandia (+Svalbard)***	***39.52***	***2.70*** × ***10^−^^17^*** ^a^
***Antarctica* versus *Cascade Mountains***	***8.651***	***1.76*** × ***10^−04^*** ^a^
Argentina versus Europe	−0.8528	0.8952
***Argentina* versus *Rocky Mountains***	***20.17***	***3.31*** × ***10^−09^*** ^a^
***Argentina* versus *Fennoscandia (+Svalbard)***	***39.52***	***2.70*** × ***10^−17^*** ^a^
***Argentina* versus *Cascade Mountains***	***8.651***	***1.76*** × ***10^−04^*** ^a^
***Europe* versus *Rocky Mountains***	***16.18***	***1.54*** × ***10^−^^07^***
***Europe* versus *Fennoscandia (+Svalbard)***	***35.36***	***1.48*** × ***10^−^^15^***
***Europe* versus *Cascade Mountains***	***5.782***	***0.001824***
***Rocky Mountains* versus *Fennoscandia (+Svalbard)***	***9.447***	***8.77*** × ***10^−^^05^***
Rocky Mountains versus Cascade Mountains	1.572	0.07192
***Fennoscandia (+Svalbard)* versus *Cascade Mountains***	***14.7***	***6.46*** × ***10^−^^07^***

Note

Significant results (bolded and italicized) indicate that these algae have genetically distinct populations.

^a^ Post hoc AD comparisons where sample comparison tests were significant, but one or more group tested had sequences counts of two or less, which may indicate low power to differentiate differences, so the veracity of these results are suspect, but included for transparency.

## 4 DISCUSSION

Here, we present an in‐depth investigation into *Sanguina* spp. snow algae biogeography. We utilize all public *Sanguina* ITS2 rDNA sequences at the time of analysis, in addition to many previously unpublished sequences. This work demonstrates that utilizing an entropy‐based approach to examine sequence variation on a sub‐OTU level can reveal previously undetected geographic and population patterns of *Sanguina*. Traditional OTU‐based clustering methods based on sequences dissimilarity have indicated that *Sanguina* populations are globally inclusive of only one to a few OTUs, suggesting no population structure (Brown & Jumpponen, 2019; Procházková et al., 2019). This is further supported by a haplotype analysis of *Sanguina nivaloides* ITS2 sequences that shows no population structure, although it does demonstrate diversity in ITS2 haplotypes (Procházková et al., 2019). Here, we demonstrate that this apparent lack of geographic population structure may be an artifact due to the relative low resolution of distance‐based OTU clustering analyses, and in fact these populations do show structure across a global landscape. Similar results from algal symbionts of coral support the idea that MED nodes provide increased ability to detect variants in the ITS2 region over traditional OTU clustering (Smith, Ketchum, & Burt, 2017). Previous work on snow algae has utilized a similar nucleotide entropy approach (oligotyping) to investigate snow and glacier algae (Lutz et al., 2018) across the Greenland Ice Sheet and also revealed algal diversity undetected by traditional OTU clustering methods, but this study stopped short at investigating spatial segregation of sequences.

Here, we confirm previously suspected Northern Hemispheric endemism of *S. aurantia*, as we found no sequences in any of the combined genetic repositories that belong to *S. aurantia* south of Colorado (USA) and expand the currently known *S. aurantia* range to include Fennoscandia, Alaska, and the Cascade Mountains. Additional sampling will likely extend this range, but it is uncertain if the true range will be extended south of the equator. Further, we detect distinct structure (different MED node distributions) of *S. aurantia* between all sampling locations (Table 2) with the exception of between Alaska and the Cascade Mountains in Washington State. This is interesting as it suggests that that the Rocky Mountains, Fennoscandia (+ Svalbard), and the Pacific Northwest (Alaska and the Cascade Mountains) have isolated *S. aurantia* populations, but this study falls short in its ability to answer if this means that there is little or no gene flow between these populations. The reasons why the Alaska and Cascade Mountain locations house similar populations is unknown. Given the vast distance between these sampling locations (~2,000 km), one might expect more population differentiation. However, these two regions have similar glaciation histories and are affected by similar atmospheric circulation patterns (Bitz & Battisti, 1999), this along with the similar refugia patterns (Geml, Tulloss, Laursen, Sazanova, & Taylor, 2010) may partially explain these similarities. It could be that these populations are remnants from the last glacier maximum (LGM) where this entire region was covered in a semi‐continuous ice sheet, but this ice sheet was discontinuous from Rocky Mountain and European ice sheets during the LGM (Ray & Adams, 2001). Thus, Alaska and Cascade Mountain populations may have only been isolated in recent geologic memory, not allowing for much divergence of the ITS2 region. Alternatively, Alaskan and Cascade Mountain populations may be part of the same metapopulation, which may explain genetic similarity, as has been documented in other algal systems (Buonomo et al., 2017). However, one of the tenants of metapopulation theory is that subpopulations have a reasonable probability of movement across the metapopulation landscape (Keymer, Marquet, Velasco‐Hernández, & Levin, 2000) and it is uncertain if *Sanguina* are capable of this movement. Future studies must examine additional loci and have expanded sampling ranges to confirm that these two locations are in fact genetically similar.

In contrast, *S. nivaloides* appears to have a global and bi‐bipolar distribution (similarly reported by Procházková et al., 2019; Segawa et al., 2018). Based on available data, populations of *S. nivaloides* exhibit spatial segregation and population structure globally with the exception of samples collected in the contiguous United States (Cascades and Rocky Mountains). Further, it appears (Figure 2) that Northern Hemisphere populations are quite distinct from Southern Hemisphere populations (with the possible exception of Argentina and Europe [Table 2] but this is only based on a single Argentinian sequence, so caution must be exercised when making inferences about these populations). This suggests long‐distance isolation by distance patterns in these snow algae (similar to Schmidt et al., 2011) but such an isolation by distance is not seen on local or regional scales (Brown et al., 2016). This is in direct opposition to previous studies that suggest a lack of global population structure of this snow alga (Brown & Jumpponen, 2019; Procházková et al., 2019; Segawa et al., 2018). Again, as with *S. aurantia*, this is likely due to the limited resolution afforded by traditional distance‐based OTU clustering methods to distinguish between populations that an entropy‐based analysis appears not to suffer from, or due to analyses based on limited sequence representation (Procházková et al., 2019). Further, while several other comparisons were indistinguishable in our *post hoc* analysis (Table 2), given that Antarctica and Argentina consisted of so few sequence (two and one sequence respectively), these population similarities should be treated with caution, but in the interest of transparency, these data are retained here. The question remains, why are populations similar between the Cascade and Rocky Mountains (~1,500 km distant) but dissimilar elsewhere? These two mountain ranges are not continuous but do have several substantial ranges in between them including the Teton, Sawtooth, and Wallowa Mountains suggesting the potential for metapopulation maintenance via the stepping‐stone hypothesis (Yang et al., 2016). Or, these populations could be a result of similar physicochemical conditions found in these two ranges. More research is needed to disentangle snow physical and chemical properties between these two sites. It remains unresolved why these two *Sanguina* species have contrasting metapopulation dynamics; *S. aurantia* populations were genetically distinct between the Cascade and Rocky Mountains whereas *S. nivaloides* were not.

Here, we used ITS2 rDNA locus‐targeted sequencing data to examine global population structure of *Sanguina* spp. which had previously been assumed to be largely nonexistent. It should be noted that the ITS2 is not a common gene target for phycological examinations but has been demonstrated to be invaluable in delineating species descriptions (An et al., 1999; Procházková et al., 2019) and for snow algae community ecological examinations (Brown & Jumpponen, 2019; Brown et al., 2015, 2016; Segawa et al., 2018). We have demonstrated that *Sanguina* populations (*S. nivaloides* and *S. aurantia*) exhibit population structure across large distances but are similar regionally. However, we lack genetic resolution to examine and elucidate mechanisms for these population differences currently. Future work should focus on expansion of sampling locations and loci sequenced (or genomic regions sequences such as whole chloroplast sequencing or genomic surveys such as RAD‐Seq) to elucidate biogeographical patterns to answer questions on dispersal ecology and gene flow between populations. Nevertheless, it is clear with this current work that these populations can no longer be considered to have a lack in geographic structure.

One of the great unanswered questions in snow algae research is how these *Sanguina* species became globally distributed and how they disperse? Once dispersed onto uncolonized snows, what are the drivers of population establishment? The latter question is beyond the scope of this work, but we can postulate on the first. There are three likely explanations (not mutually exclusive) on how snow algae disperse: (a) animal vectors, (b) aerial transport, and (c) legacy effects from previous establishments. The most discussed option for animal transport is via fecal droppings of birds or insects (Kristiansen, 1996; Proctor, 1959; Revill, Stewart, & Schlichting, 1967; Schlichting, 1960). However, this seems unlikely for long‐distance transport as insects generally do not move on the scale of intercontinental transport and most long‐distance migratory birds exhibit atrophy of the digestive system during migrations which limits aerial waste release (McWilliams & Karasov, 2005). Additionally, snow algae could rely on aerial transport. However, long‐distant transport may be unlikely outside of extreme weather events (Brown & Jumpponen, 2019) due to the relative large size of their hypnozygotes (*ca*. 20 μm in diameter); propagating units of this size are only modeled to be capable of atmospheric transport for around 12 hr (Wilkinson, Koumoutsaris, Mitchell, & Bey, 2012), and the limited published aerial sampling for snow algae has revealed no appreciable algae cysts (Novis, 2001). However, there is evidence of mid‐range aerial dispersal of Antarctic algae (Marshall & Chalmers, 1997). *Sanguina* hypnozygote morphology may provide the answer; when hypnozygotes are slightly desiccated, *Sanguina* may have raised veined ridges (see figure 5c in Procházková et al., 2019) which may assist aerial transport. This is reminiscent of echinolophate pollen morphology in some Compositae plant species that is hypothesized to aid in long‐distant transport (Bolick, 1978; Keeley & Jones, 1979). Of course, any apparent morphological similarity may be inconsequential but together, this suggests that aerial dispersal may be viable mode or organismal transport, but likely not on an intercontinental scale, and additional work is needed to confirm this capability.

Populations might also be the result of legacy effects from previous global snow and glacier algal colonization. Snow communities, and algae in particular, may have played a large historic role during the Cryogenian (720–635 MYA), a period marked by extreme cold and near‐global glaciation. Geologic records and modeling to this end suggest cold‐tolerant algae were the dominant primary producers during the Cryogenian, sequestering massive stores of organic carbon that subsequently released when the climate warmed (Hoffman, 2016). We may find that current extant populations may be remnant populations of this historic radiation, but dated phylogeographic analyses to confirm this are not feasible with current data. Based on models of snow cover during that period (Hoffman et al., 2017), snow algae potentially covered the majority of Earth's habitable surface area and may have influenced snow melt rates and movements of organic carbon pools (Ganey, Loso, Burgess, & Dial, 2017; Hood, Battin, Fellman, O'Neel, & Spencer, 2015). However, it remains unresolved if contemporary snow algae are as influential to global or local nutrient cycling dynamics (but see Hamilton & Havig, 2020).

Here, we suggest that snow algae within *Sanguina* do not have homogeneous population structure across their respective ranges as has previously been suggested. The discrepancy between our results and those previously reported is likely due to the hypersimilarity of the ITS2 region of *Sanguina* species that traditional distance‐based OTU clustering analyses are unable to resolve. Instead, we see that *S. aurantia* has a circumpolar and temperate alpine distribution in the Northern Hemisphere with largely distinct population structures and *S. nivaloides* exhibits bipolar and alpine distributions that are broadly genetically distinct. This represents a novel understanding of *Sanguina* distributions and highlights that there is a dearth of information about these snow algae, illustrating the need for additional investigations to fully characterize their biogeographic and evolutionary histories. Given that these algae are only known to exist in the rapidly disappearing and endangered cryosphere (Derksen & Brown, 2012), detailed genomic, phylogeographic, and ecological studies are desperately needed to understand this unique aspect of biodiversity before it disappears entirely.

## CONFLICT OF INTEREST

The authors declare no conflicts of interest.

## AUTHOR CONTRIBUTION

**Shawn Brown:** Conceptualization (lead); Data curation (lead); Formal analysis (lead); Funding acquisition (lead); Investigation (lead); Methodology (lead); Resources (lead); Software (equal); Supervision (lead); Validation (lead); Visualization (equal); Writing‐original draft (lead); Writing‐review & editing (equal). **Avery Ezra Tucker:** Conceptualization (supporting); Software (supporting); Visualization (supporting); Writing‐original draft (supporting); Writing‐review & editing (equal).

## Supporting information

Appendix S1

Table S1

## Data Availability

All sequences used and analyzed here are publicly available from SRA, GenBank, or supplemental information of referenced papers. All sequences analyzed are also available as an Appendix and archived in Dryad at https://doi.org/10.5061/dryad.pzgmsbcj3.
